# The Condition of the Fœtal Circulation in New-born Infants

**Published:** 1853-07

**Authors:** 


*The Condition of the Fœtal Circulation in New-born Infants.*—Dr. Elsasser, of Stuttgard, after 370 examinations of the fœtal bloodvessels, has arrived at the following conclusions: Of 70 stillborn children, the tubes peculiar to the fœtal circulation were open in 69. In one only, the ductus botalli and the foramen ovale were open and the ductus arantii closed. In 300 children who died soon after birth, 80 out of 108 prematurely born, and living from one to eight days, presented all the passages open; 127 out of 192 infants of the full time had all the passages open, but partly contracted.

The ductus botalli (arteriosus) was open 55 times, and completely closed 10 times; the foramen ovale open 47 times, completely closed 18 times; the ductus arantii (venosus) open 81 times, completely closed 37 times. These facts prove that the vessels peculiar to the fœtal circulation remain open, as a rule, for some time after birth, and that it is not possible to determine accurately by days the period of their closure.

The process of obliteration pursues the following order as regards time: It commences and is often completed in the ductus venosus, before it manifests itself in the other vessels. There are great varieties in the closure of the foramen ovale and ductus arteriosus. The completion of the process is, in by far the greater number of cases, within the first six weeks; and the instances of obliteration of this or that duct before birth, or before four to six weeks after birth, are exceptions, of mostly forensic interest. The ductus arteriosus exhibits the process of obliteration by the wrinkling and swelling of the inner vascular coat, commencing usually at the aortic extremity; most rarely at the pulmonary end. In the ductus venosus, the point of commencement is usually towards the vena cava; sometimes towards the umbilicus; most rarely in the middle. The blood coagulates in the ductus arteriosus; the filling up of the tube evidently comes from the thin, smooth lining membrane of the vessel; a structure, resembling an adventitious membrane, may be easily peeled off; it is mixed with blood disks and fibrinous coagula.—*Henle's Zeitcsh.* 4, 1852.

